# Honey and Its Antimicrobial Properties: A Function of a Single Component, or the Sum of Its Parts?

**DOI:** 10.7759/cureus.17718

**Published:** 2021-09-04

**Authors:** Steven Sartore, Seth Boyd, Daniel Slabaugh, Nikhil Jain, Blake Piepenbrink, Stephanie Blount, Zimrisha Alla, Walters Cheso, Hunter Belanger, Thomas P Arnold

**Affiliations:** 1 Research, Lake Erie College of Osteopathic Medicine, Bradenton, USA; 2 Research, Lake Erie College of Osteopathic Medicine School of Pharmacy, Bradenton, USA; 3 Medicine, Lake Erie College of Osteopathic Medicine, Bradenton, USA

**Keywords:** honey, antimicrobials, protein extraction, lipid extraction, disc-diffusion, apidaecins

## Abstract

Introduction: Honey is known for exhibiting antibacterial properties, indicating its use as part of traditional medicine since the early ages. With the advent of antibiotic-resistant bacteria, the need for alternative antimicrobials has outpaced the actual development of novel, broad-spectrum antibiotics. Previous research has revolved around the sugar content of honey because its sweetness makes it an attractive food source. However, research assessing the protein and lipid components of honey is lagging behind that of its sugar counterpart. The goal of this investigation was to examine the antimicrobial properties of honey and to identify any distinct proteins or lipids.

Methods: In order to isolate individual peptides and lipids, the different samples of local and foreign-sourced honeys were dialyzed, and the resulting dialysate proteins were screened via gel electrophoresis (sodium dodecyl-sulfate polyacrylamide gel electrophoresis [SDS-PAGE]) with Coomassie blue and silver stain, while lipids were examined using thin layer chromatography (TLC). To assess antimicrobial potency, a series of Kirby-Bauer disc diffusion assays was performed on Mueller-Hinton agar using different types of raw honey with *Staphylococcus aureus*, *Staphylococcus epidermidis,* *Pseudomonas aeruginosa*, *Escherichia coli*, and *Bacillus subtilis*. The process was then repeated using the peptide extracts from the dialyzed fractions of the honeys.

Results: The SDS-PAGE trials revealed repetitive promising protein bands across several gels below 75kDa with both Coomassie blue and silver staining. The TLC analysis of varying raw honey samples consistently demonstrated the presence of medium and long-chain fatty acids, likely in the range of C12-C14. In the disc diffusion assays, the greatest amount of inhibition was seen when the honeys were tested as a whole instead of its constituent parts.

Conclusion: Instead of an individual component acting as the key to honey’s action against bacteria, it appears there is a synergistic relationship amongst the sugars, proteins, and lipids that make each honey unique.

## Introduction

Honey has been used as a cultural remedy for a variety of ailments and some research has recently demonstrated promise as an antimicrobial. It is a natural compound produced by bees via nectar from flowers. The natural production of honey by bees is unique in that the contents of honey vary depending on the species of bee and flower as well as the season the honey was produced [1,2]. Several studies have examined whole honey’s ability to counteract bacterial growth compared with hypertonic sugar solutions, and the honey samples proved to be more effective than the sugar controls [3]. Specifically, bees have been shown to produce a diverse array of honeys with proteins called apidaecins [4]. These proteins are significant because they have been shown to have antibacterial properties against gram-negative bacteria including *E. coli* and *P. aeruginosa *[4,5]. Apidaecins act as a part of the immune system of the bees. Apidaecins, while distinct from antibodies, act in a similar manner by undergoing changes to the variable regions, allowing them to target different bacteria species [4].

As noted, honey production is highly unique due to variations in bee species and flower type. However, it is also observed that the sourcing of honey can also play a role in its composition, specifically looking at locally sourced honey versus commercially produced honey [5]. Research has shown that biological and chemical profiles can be significantly different between local and commercial honey. Some of the differences proposed by Šedík et al. suggest that locally produced honeys have greater antioxidant and antimicrobial activity. Interestingly, the research also shows mineral composition differences with the primary element in local and commercial honey being potassium and sodium, respectively. Geographical location of honey production also influences differences in phenolic compound profiles [6]. Phenolic compound variety is notable given their role as an antioxidant in honey.

There is currently limited research available that examines the mechanism behind this antibiotic action. In order to elucidate any antimicrobial agents responsible, honey must be separated into its macromolecules and then exposed to various organisms. Current methods for protein extraction from honey include isolation via dialysis and lyophilization, as well as protein dialysis with precipitation [2,7]. Dialysis is a process in which a semipermeable membrane is used to separate proteins based on their size. A dialysis tube with the protein-containing solution is used to allow small molecules to go from an area of high concentration to low concentration and pass through the membrane, leaving the large molecules, or proteins, inside the tubing. Following dialysis, proteins can be further isolated through lyophilization or precipitation [7]. Lyophilization, or freeze-drying, is a process that involves freezing the solution followed by the addition of heat through a vacuum. This causes the ice to sublime, and the water is then removed through the process of desorption. While numerous techniques are available, extracting sufficient amounts of protein from honey can be problematic due to the low fraction of protein present in honey [2].

The goal of this research was twofold: to find an effective way to isolate proteins and lipids from honey and to examine these individual honey components for possible antimicrobial factors or activities.

## Materials and methods

Honey selection: 

With hundreds of honey species available, it was necessary to reduce the number of analyzed samples to a manageable size. A thorough investigation of literature yielded two honeys that had previously been associated with apidaecins and antimicrobial activity, the Manuka and Slovakian varieties. These two honeys with their bacteriostatic and bactericidal activities were compared to a honey sourced from a local beekeeper, named Carter’s Honey™ (Bradenton, FL, USA) in addition to several other regional species. Further, the various honey samples were used for lipid and protein analysis. Honey samples were donated by the Suncoast Beekeepers Association or purchased at a local store. 

Protein extraction: 

Dialysis and precipitation methods were used to extract proteins from the whole honey samples. Separate dialysis tubes with cutoffs of 3500 Daltons (Da), 8000 Da and 14000 Da were filled with 25 ml of whole honey sample. The tubes were placed in a flask with 1.5L of solution consisting of 1350ml of water and 15 ml of 9% saline for a final concentration of 0.9% NaCl. 

The tubes remained in the solution for 24 hours until the solution became saturated with sugars. The solution was then replaced with another 1350ml of water and 150ml of 9% saline and the process was repeated. Eight milliliters of the remaining solution in each dialysis tube was combined with 40ml of acetone and placed in a freezer for 24 hours to precipitate the proteins. The acetone was removed, and the remaining protein isolates were solubilized and used for the Kirby-Bauer disc diffusion assay. 

Protein identification: gel electrophoresis 

Gel Prep

A two-level gel was made in the lab with the lower gel consisting of 4.8mL water, 2.5mL 4X lower tris, 2.5mL of 37.5:1 acrylamide, 100uL of 10% ammonium persulfate (APS) and 15uL of tetramethylethylenediamine (TEMED). The upper gel consisted of 2.9mL water, 0.5mL acrylamide, 0.5mL upper tris, 50uL APS and 10uL TEMED.

Sample Prep and Loading

The samples for each lane were a 2:1 ratio of dialyzed honey to sodium dodecyl-sulfate (SDS) sample buffer, respectively. 3,000 Da, 8,000 Da, and 14,000 Da dialyzed honey samples were given two lanes for each category: one larger total volume (60uL total) and one of half of that volume (30uL). The last lane was loaded with dialysate (the fluid the dialysis tubing was submerged in) to assess if proteins were able to diffuse.

Running, Staining, and Destaining

The gels were run at 80 volts over the course of three hours in a Biorad® Protean III cell. The gel was stained with either a Coomassie Blue dye or silver stain for at least 12 hours and destained with a small amount of destaining solution (300mL DI water, 150mL methanol, and 50mL acetic acid). The gel preparation, running and staining were performed in triplicate fashion.

Lipid identification: thin layer chromatography 

For each dialyzed honey sample, 10mL of each sample was pipetted into a centrifuge tube, then 10mL of hexane was added to separate the proteins from the lipids. Each tube was then vortexed for 30 seconds and the supernatant was pipetted out. The centrifuge tubes were placed in a fume hood overnight, allowing the remainder of the supernatant to evaporate. Next, 50uL of 10% Triton was added to each vortex tube. It was vortexed for 15 seconds and then any remaining liquid was pipetted out. The lipids were now concentrated, and capillary action tubes were used to spot the TLC plates. The study had 10 different lanes, three of which were of the concentrated honey lipids from each dialysis cutoff, one was of a raw honey sample, and the others were of lipid standards. The standards included stearic acid, palmitic acid, palmitoleic acid, squalene, wax, and neatsfoot oil (a mixture composed of bovine skin lipids). The TLC plate was then placed into a chamber with a solvent consisting of chloroform:methanol:water (60:30:5 by volume), once the solvent rose halfway up the plate, the TLC plate was then moved into a different chamber with a solvent consisting of hexane:petroleum ether:acetic acid (80:20:1.5 by volume). Once the solvent rose to near the top of the plate, the TLC plate was placed into a third chamber consisting of iodine gas. It was taken out of the iodine tank and exposed to UV light for best visualization of lipid compounds. The TLC analysis was repeated twice for each honey sample.

Antimicrobial susceptibility: Kirby-Bauer disc-diffusion assay 

The antimicrobial effects of the whole honey samples and isolated protein extracts were assessed using Kirby-Bauer disc diffusion assays. Mueller-Hinton agar was used to culture *S. aureus*, *S. epidermidis*, *P. aeruginosa*, *E. coli*, and *B. subtilis*. Ten microliters of either the whole honey sample or proteins extract was pipetted onto a disc. The discs were placed on Petri dishes with Mueller Hinton agar and the cultured bacteria. The Petri dishes were then placed in an incubator at 37°C. After 48 hours, the Petri dishes were removed, and the maximum diameter of each zone of inhibition was then measured and recorded in centimeters. The honey samples were compared to a control disc of 30µg tetracycline. All assays were repeated in triplicate and their average zones of inhibition were recorded.

## Results

Protein and lipid analysis

**Figure 1:**
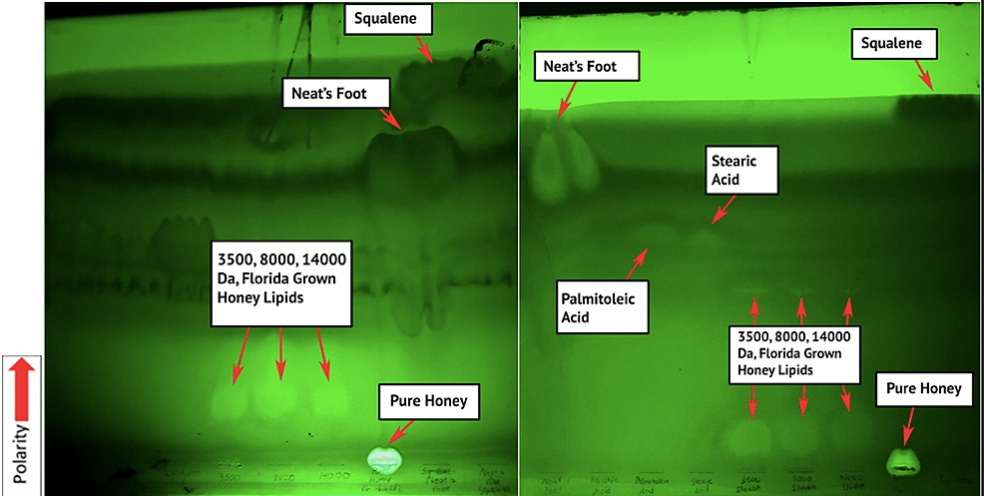
An example of the thin layer chromatography (TLC) plate spotted with local Florida-grown honey (left) and Slovakian honey (right) under 365nm UV light.

**Figure 2:**
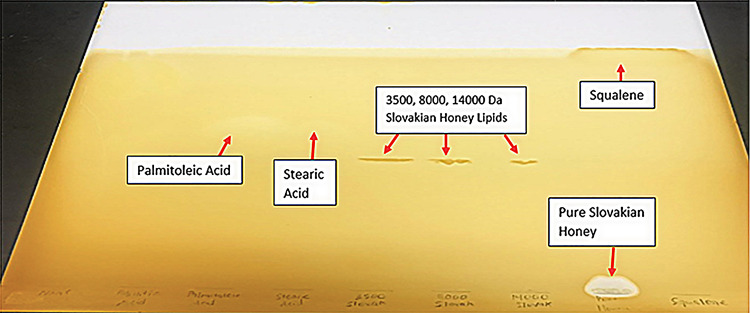
Thin layer chromatography (TLC) plate spotted with Slovakian honey after being exposed to iodine gas tank.

TLC plating revealed large bands of lipids near the bottom of the plates for all three dialysis cutoffs (Figure 1). These bands were best visualized before being placed in the iodine tank and using UV light at a wavelength of 365 nm. Small bands were also noted halfway up the plate for all three dialysis cutoffs. These were best visualized after being placed in the iodine gas tank (Figure 2) but could also be seen using UV light (Figure 1 - right). Both figures represent the results seen with each repetition of the analysis.

**Figure 3:**
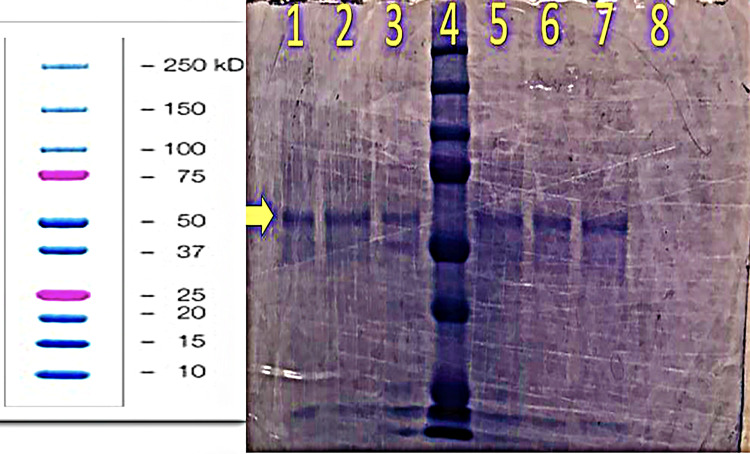
Lane 1: 40uL 3500Da dialyzed honey to 20uL SDS, Lane 2: 20uL 3500Da dialyzed honey to 10uL SDS. Same dilution pattern in lanes 3 & 5 with the 8000Da dialyzed samples and lanes 6 & 7 with the 14000Da dialyzed samples. Lane 4: Biorad© Precision Plus Protein standard (10-250kD). Lane 8: 14,000Da dialysate. Bands (arrow) lanes 1-7 around 10, 15 and 60kDa. Left side of Figure 3 - Biorad Precision Plus Protein ladder reference SDS: sodium dodecyl-sulfate

As seen in the representative example of the electrophoresis gels in Figure 3, there is a strong band in all sample lanes at just above the 50kD marker (arrow). Interestingly, this band is steady in each lane, despite some lanes containing half of the amount of honey sample volume. 

**Figure 4:**
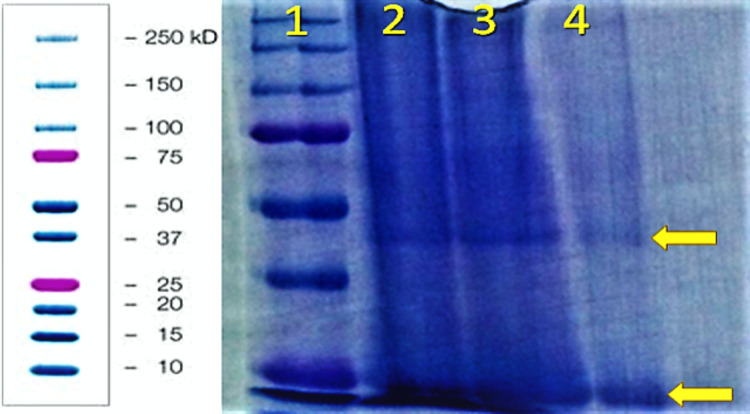
Lane 1: Biorad Precision Plus Protein standard ladder (10-250kD). Lane 2: 40uL 3500Da dialyzed honey to 20uL SDS buffer, Lane 3: 40uL 8000Da dialyzed honey to 20uL SDS buffer. Lane 4: 40uL 14000Da dialyzed honey to 20uL SDS buffer. Lanes 2-4 have bands ~40-50kD (top arrow) as well as bands ~25kD (bottom arrow) SDS: sodium dodecyl-sulfate

**Figure 5:**
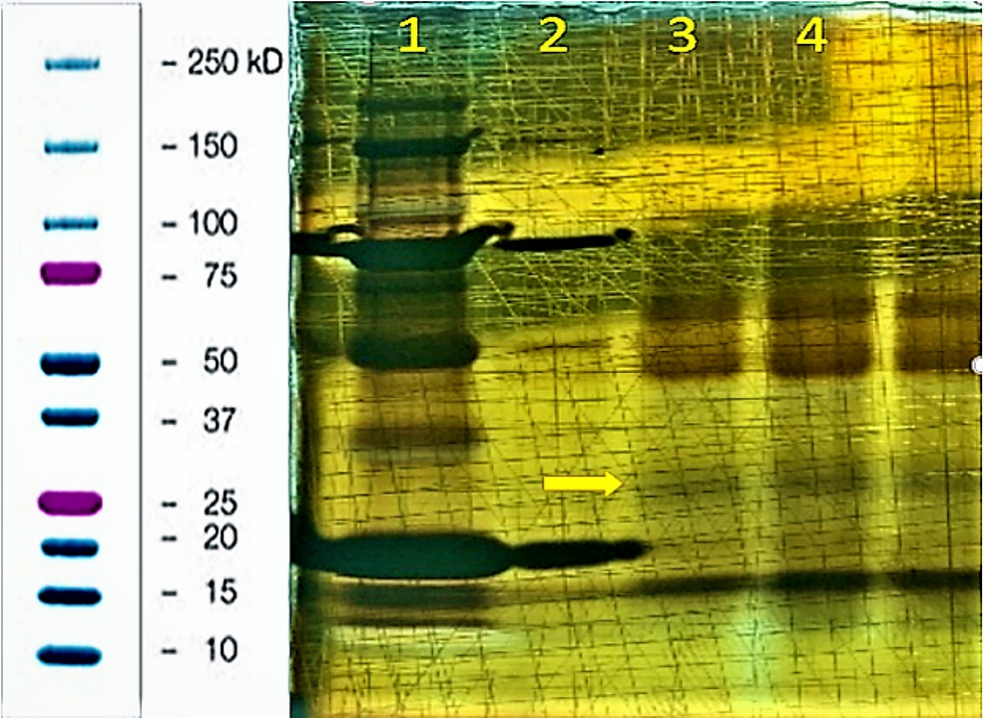
Lane 1&2: Biorad Precision Plus Protein standard ladder (10-250kD). Lane 3: 40uL 3500Da dialyzed honey to 20uL SDS buffer, Lane 4: 20uL 3500Da dialyzed honey to 10uL SDS buffer. Noteworthy bands ~25kD in lanes 3 & 4 (arrow). SDS: sodium dodecyl-sulfate

Figure 4 and Figure 5 show the surprising presence of more proteins than anticipated noted in each protein analysis trial. Figure 4 shows bands just below the 50kD marker when stained with a Coomassie Blue staining agent, and Figure 5 showing protein bands in multiple lanes just above the 25kD marker when stained with a silver staining agent.

Antimicrobial analysis

**Figure 6:**
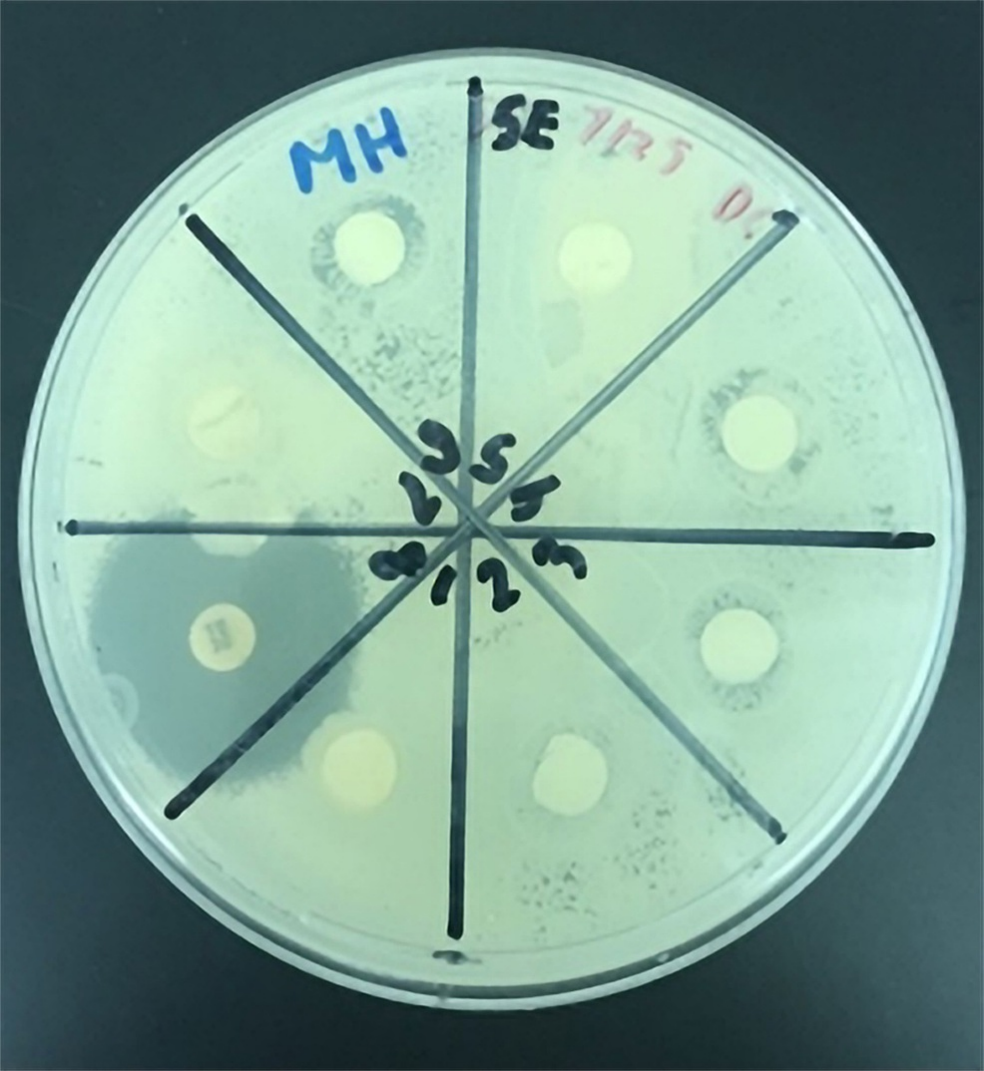
Kirby-Bauer disc-diffusion assay demonstrating the antimicrobial activity and resulting zones of inhibition (ZOI) of whole honey samples when plated against S. epidermidis. *Each zone of inhibition (ZOI) diameter measured in centimeters (cm) - Disc 1:* James Cutway Honey - 0cm *Disc 2: *Myakka River Honey - 1cm *Disc 3:* Slovakian Honey - 1.2cm *Disc 4:* Manuka Honey - 1.2cm  *Disc 5: *Stephanie Suter's Honey - 0cm  *Disc 6: *Carter's Honey - 1.3cm  *Disc 7: *Nigeriain Honey - 0cm  *Disc 8: *30ug tetracycline standard - 2.9cm

**Figure 7:**
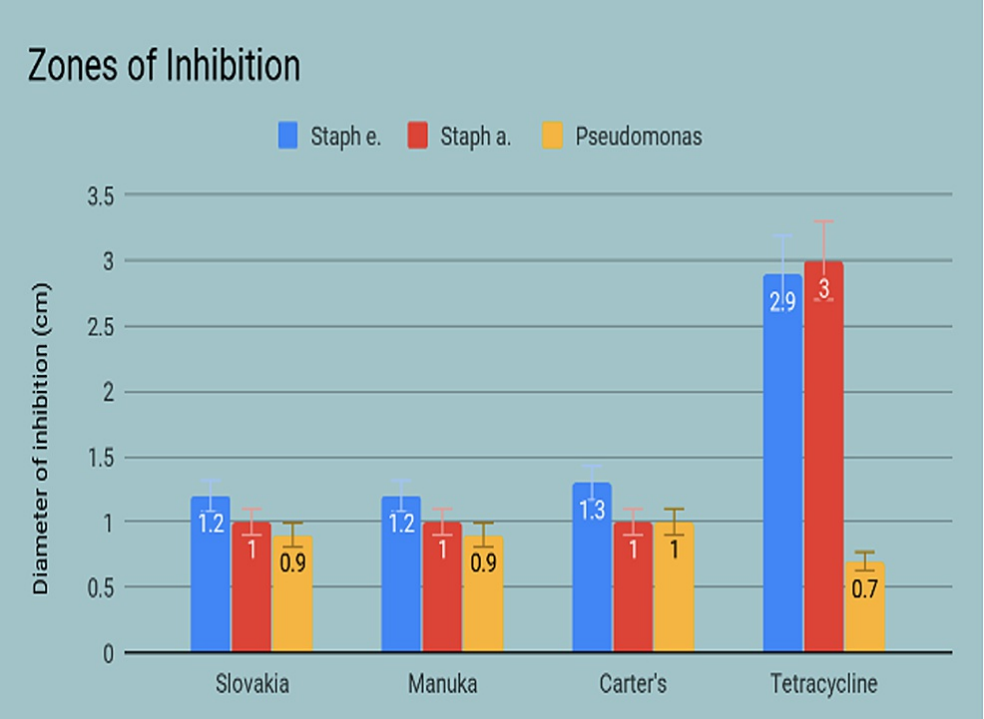
Average zones of inhibition measurements of the whole honey samples with highest antimicrobial activity.

**Figure 8:**
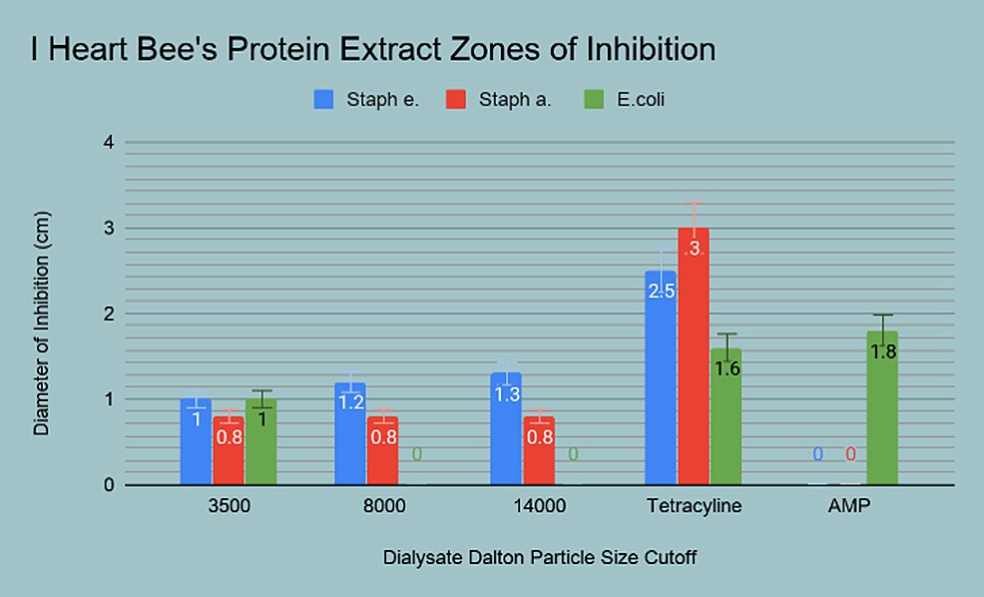
Average zones of inhibition measurements of the protein extract from the I Heart Bee’s™ honey sample.

The whole honeys sampled were compared head-to-head using the Kirby-Bauer disc diffusion assays and their respective zones of inhibition were measured, an example of which is shown (Figure 6). The honey with the greatest zones of inhibition (ZOI) against all three strains of bacteria was Carter’s Honey™ from Bradenton, Florida. This was compared to the Manuka and Slovakian species that also displayed antimicrobial activity; however, the remaining whole honey samples examined did not display measurable inhibition and were therefore excluded in the final analysis (Figure 7). Additionally, the extracted proteins from I Heart Bee’s™ (Polk City, FL, USA) local sourced honey exhibited a marginal amount of inhibition against the prominent strains tested (Figure 8).

The results of this study reveal a difference between whole honey and its concentrated lipids and proteins in the ability to inhibit bacterial growth. Further statistical analysis is required to determine whether the results observed are significant. Out of the macromolecules tested, the protein extract appeared to have the strongest influence on the antibacterial properties of the whole honey sample. Despite this apparent effect, the average zone of inhibition was larger for the whole honey samples compared to the concentrated proteins. 

## Discussion

In the face of antibacterial resistance, new antimicrobial compounds must be investigated. One particular avenue, alternative medicine, may provide some clues on this search. The present study examined one such modality with proposed benefits: honey. Several lipids and proteins were isolated and identified, with the protein extracts undergoing further evaluation for antimicrobial activity. Honey’s antibacterial effects as a whole product were also monitored and compared to its separated constituent macromolecules. 

**Figure 9:**
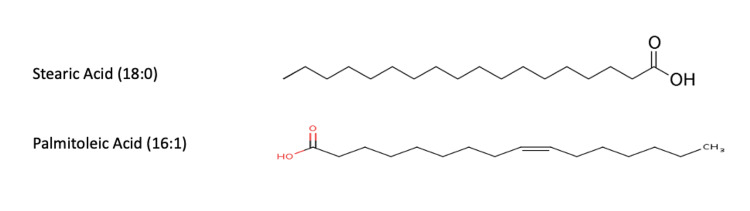
Carbon structure of the palmitoleic acid and stearic acid standards.

It is believed that the large bands of lipids at the bottom are a milieu of proteins and lipids that were unable to separate during dialysis or the use of hexane or Triton. The charges of the proteins keep the lipids at the bottom of the plate because they have a strong affinity for the first polar solvent, chloroform:methanol:water. It most closely resembles the raw honey sample that contains numerous proteins and sugars that predictably have an even greater affinity for the first polar solvent causing it to not move. The small bands halfway up the plate likely indicate the presence of medium-chain fatty acids in the range of C10-C12. This is inferred from the fact that these bands are below the bands from the palmitoleic acid and stearic acid standards, which are C16:1 and C18:0 fatty acids, respectively (Figure 9). They had the greatest affinity for the second, less polar solvent, hexane:petroleum ether:acetic acid, while the most nonpolar fats such as neatsfoot oil and squalene rose to the top. It is hypothesized that these medium-chain fatty acids are some of the precursor components used by bees to make beeswax by esterification.

In reference to the proteins seen in the gels of Figures 5, 6, and 7, it is clear that a diversity of proteins partially constitute honey. Interestingly, in a study by Sahlan et al. using an Indonesian honey known as Tertragonula sp., three protein bands were found (52.96-61.9kD, 63.35-65.92kD, and 86.16-91.4kD) when run on gel electrophoresis [8]. The prominent and reliable bands seen in Figure 5 fall in the 63.35-65.92kD range, showing promising consistency with the results obtained by Sahlan et al. This previously published evidence on the Indonesian honey prompts enthusiasm and foundation to send this specific protein band/extraction for protein sequencing. Further evaluation using bioinformatics tools could even yield identifiable structural patterns, usable for antibiotic production.

It was noted in this trial that honey had exhibited a higher antibacterial effect on certain bacteria species. In particular, the whole honey sample from Carter’s Honey created a larger ZOI against *Pseudomonas* than the tetracycline control. However, all three of the honey samples lacked equivalent inhibition as tetracycline when plated with *S. epidermidis* and *S. aureus*. Although not as effective as conventional antibiotics, the honey samples demonstrated the antimicrobial activities that had been previously described in the literature. This consistent bacteriostatic activity may make honey a suitable adjuvant therapy to conventional antibiotics. The strains of *S. aureus* and *S. epidermidis* were most susceptible to both the whole honey samples and protein samples, which suggests that the antibacterial properties of honey may be more effective against these Gram-positive organisms. This notion is supported by Cooper et al. who found that regardless of antibiotic sensitivity, gram-positive bacteria were equally inhibited by Manuka and pasture honeys [6,7]. Despite a clear mechanism as to honey’s activity against these strains, the study reiterated the actions are more intricate than previously assumed. Our study results corroborate that the whole honey inhibits bacteria by mechanisms other than osmolar dysregulation. Additionally, it was noted the whole honey samples displayed similar ZOIs as the protein extract against *S. aureus *and *S. epidermidis*. However, the difference between these means was not analyzed and therefore cannot be deemed statistically significant. This presents the opportunity for further exploration into the antibacterial qualities of honey proteins. The lipid fractions isolated from the honey samples were not used in the antimicrobial assay, offering another aspect of this research to be expanded upon. The whole honey samples and protein extract displayed encouraging antibacterial properties, which furthers the possibility of a synergistic relationship between the individual macromolecules.

It is evident that certain honeys had more potent antimicrobial effects than others. That is no surprise given the diverse number of bee species and honeys they produce. Although a diverse population of honeys could have been examined, our study focused on the few species that were readily available as well as locally sourced. By keeping the variety of tested honeys to a small sample size, the study could be conducted more judiciously and thoroughly investigated. The natural limitation of our sample size rests on the possibility of more potent antimicrobial properties in the hundreds of honey varieties that were not dissected or tested. Future research utilizing a similar methodology as the one described here on a larger cohort of honeys may provide an enhanced view of a particular honey’s components. Further trials of the disc-diffusion assay should be completed in order to add significant power to our results, which were limited due to a small sample size. For honeys that continually exhibit bacterial inhibition, those varieties may undergo protein analysis to explore if the protein structures vary among exceptionally antimicrobial honeys. Additionally, other bacteria that cause skin infections like methicillin-resistant *S. aureus* (MRSA), *S. pyogenes*, and *P. multocida* should be tested in further studies. With our results showing certain honeys have antimicrobial properties especially against Gram-positive organisms, it is an exciting possibility that honey could be used as a vehicle for transmission of antibiotics into the epithelium to combat skin infections. Honey could potentially have strong enough effects to combat skin infections and be used as a stand-alone topical gel. This would help in the continual battle to combat antibiotic resistance. 

Further studies should attempt to utilize high-performance liquid chromatography (HPLC) to provide more detail on the lipids found in honey. Additionally, the methods to isolate proteins discussed earlier should be used to sequence any proteins to determine their biochemical structure. Supplementary research for prospective experiments can explore comparative studies of lipid compounded vs. honey compounded antibiotics and examine their respective effects on bacteria. The ability to identify the protein’s amino acid sequence and secondary, tertiary, and potentially quaternary structures could clue future researchers in on how proteins demonstrate an antimicrobial effect. 

## Conclusions

This research has shown that honey has the potential to lead to the development of new antibiotic regimens that can address the growing challenge of resistant strains. In this particular study, it was evident that there is not an obvious individual component of honey that allows it to inhibit bacterial growth. Instead, its bactericidal properties rely on the sum of its parts. By expanding upon this research and using protein sequencing and HPLC, one can obtain an even better understanding of the proteins and lipids in honey. There also remains much opportunity to Improve methods of extracting lipids from honey and continuing to utilize HPLC to obtain quantitative data of the fatty acids and other lipids present in honey. Future research can explore reasonable approaches to utilizing honey in a clinical context as a method of preventing or treating common bacterial infections. Additional trials with different concentrations of honey extract would clarify the findings of this testing period and provide confirmation about Manuka honey’s capabilities. The differences observed in the antimicrobial properties of whole honey and the extracted proteins may possibly be statistically significant. As such, there is a call on future research in the realm of statistics aimed to formally analyze the totality of experimental data collected here in order to determine whether the differences in antimicrobial properties are statistically significant. Additional experimentation related to this research project is encouraged to determine whether the conclusion of the project is accurately reproducible and empirically valid. The prospects for honey are promising. With a more refined procedure, the sign of potential seen in this primary investigation could be brought into full view for the scientific community to explore. 
